# The Simulation Analysis and Experimental Study on the Temperature Field of Four Row Rolling Bearings of Rolling Mill under Non-Uniform Load Conditions

**DOI:** 10.3390/s24030914

**Published:** 2024-01-31

**Authors:** Jianliang Sun, Hesong Guo, Xin Guo, Chao Ma, Yan Peng

**Affiliations:** 1 School of Mechanical Engineering, Yanshan University, Qinhuangdao 066004, China; ghs1993@163.com (H.G.); guoxin7707@hotmail.com (X.G.); machao312@stumail.ysu.edu.cn (C.M.); pengy516@163.com (Y.P.); 2 National Engineering Research Center for Equipment and Technology of Cold Rolled Strip, Yanshan University, Qinhuangdao 066004, China

**Keywords:** four-row cylindrical roller bearing, finite element method, temperature field, load distribution

## Abstract

As a key component of the rolling mill, the four-row cylindrical roller bearing (FCRB) operates under complex working conditions of high speed, high temperature, and heavy load. Due to the lack of an effective temperature test scheme for rolling mill bearings, a too high temperature can easily lead to bearing failure or damage under unsteady conditions. To reveal the internal temperature distribution law of four-row roller bearings of rolling mills and solve the common problem of difficult temperature monitoring of rolling mill bearings, in this paper, a four-row cylindrical roller bearing of 1140 mm cold rolling six-high mill is taken as the research object, and the temperature field calculation model for four-row cylindrical roller bearings is established. Firstly, the mechanical analysis model of FCRB is established on the basis of single row bearing by slice method. Secondly, the mechanical calculation model of FCRB is established by the Newton–Raphson method (NRM) and the finite element method (FEM). Thirdly, based on the mechanical calculation model, the finite element method is used to establish the temperature field model of FCRB under uniform load distribution and non-uniform load distribution. Finally, the temperature test experiment is carried out with the FCRB in the rolling mill fault diagnosis test bench. The results show that the error between the FCRB temperature calculation model and the experimental results is less than 10%. It can be seen that the uneven temperature distribution of FCRB is mainly caused by the uneven load distribution. The temperature distribution along the axial direction of the bearing is related to the load distribution of each column, while the circumferential temperature distribution is related to the azimuth angle.

## 1. Introduction

The four-row cylindrical roller bearing (FCRB) is a key component of cold rolling mill equipment. In the working process, it is affected by high speed, heavy load and other factors, which produce a lot of heat [1]. In the course of operation, the bearing is often burned and damaged by overheating due to insufficient oil supply or excessive rolling force [2,3]. An increase in temperature may cause lubrication failure inside the bearing, aggravate the bearing wear, and even lead to the occurrence of locking of the bearing and the reduction of the bearing clearance, which seriously affects the useful life of the bearing [4,5,6,7]. In addition, the uneven load distribution of rolling bearings during operation leads to undesirable vibration and uneven temperature of the equipment. Uneven load and uneven temperature will lead to local thermal expansion of the bearing and significant contact pressure stress, which will further generate frictional heat, a phenomenon called thermoelastic instability (TEL), which can cause serious damage to the bearing [8]. This phenomenon of uneven load distribution is common in the operation of rolling bearings [9], high-speed rotors [10,11,12], and motor spindles [9,13,14]. Therefore, it is essential to study the bearing temperature. By studying the temperature, the temperature distribution inside the bearing can be analyzed, and then the performance of the bearing can be improved through structural design, optimization of lubrication conditions, material improvements, and other methods.

At present, the temperature calculation of the bearing is mainly built up by the thermal resistance method and FEM. The thermal resistance method discretizes the entire model into multiple thermal nodes and establishes a heat balance equation within each node. The heat generated in the node equals heat dissipation, and the nodes are connected by thermal resistance. The temperature value of each knob is obtained by tackling the heat equilibrium condition of each node [15]. Harris and Kotzalas [16] calculated the temperature inside the bearing using the thermal resistance method and classified the heat transfer of the bearing. Yan et al. [17] built up the transient thermal network model of the complex construction of spindle-bearing orderliness. The influence of time-varying parameters such as deformation and viscosity on bearing temperature is considered in transient analysis. Zheng et al. [18] optimized the partition scheme of the thermal network to make the thermal network model closer to the real value. However, the transient temperature field tends to become complicated when the parameter changes are considered, and it is difficult to effectively predict the internal temperature of the FCRB of the rolling mill under the condition of alternating loads. Furthermore, the thermal resistance method studies rolling bearings in two-dimensional space. Although this method is simple and convenient, it cannot fully reflect the temperature of the bearing at different azimuth angles and has certain limits in the temperature examination of rolling bearings. The FEM is similar in principle to the thermal resistance method, and both need to calculate the temperature production and heat dissipation of the rolling bearing. Although the FEM is more complicated in the process of solving the bearing temperature field, it can effectively reflect the temperature inside the rolling bearing and has certain advantages in analyzing the temperature field of a specific three-dimensional model. Deng et al. [19] explored the temperature calculation model of the spindle-bearing system, which has high computational efficiency and accuracy. Aleksandar et al. [20] studied the internal temperature of high-speed spindles, using a temperature computation model that reflects the temperature differences of ball bearings at different locations. They analyzed the reasons for the temperature differences and provided a reference for establishing a temperature computation of non-uniform load cylindrical roller bearings. In addition, restricted by the narrow space inside the bearing structure and the high-speed rotation state, Zhang et al. [21] prepared an improved quantum dot sensor by using the characteristics of small size, high precision, and being unaffected by the rotation factor of the quantum dot sensor, so that the bearing rotating element can carry out thermal monitoring under extreme working conditions. The team [22] used CdTe quantum dot sensors for the first time to measure the temperature of the bearing cage and inner ring at speeds of 5000–6000 r/min, and conducted research on the thermal characteristics of the bearing inner ring cage. Kim et al. [23] built up a temperature computation model of ball bearings using the FEM, but the model could not reflect the temperature spreading of the external ring well. Zhou et al. [24] used the APDL module to analyze the fever of the ball bearing. They combined it with the experiment to measure the temperature of each node inside the bearing, revealing the temperature distribution in the bearing. Yan et al. [25] modified the thermal coefficient of high-speed rolling bearings and verified the necessity of thermal coefficient correction.

The current thermal analysis of bearings mainly focuses on single-row ball bearings, and there is still a lack of theoretical analysis for the temperature field of FCRB, especially FCRB under uneven loads. In fact, the temperature non-uniformity of the FCRB caused by the non-uniform load is very serious. Therefore, the temperature analysis of FCRB in rolling mills is very important. In this paper, the FCRB of a 1140 cold rolling six-high mill is taken as the research object. The main contributions of our paper can be summarized as follows:

(1) Based on the mechanism and mechanical model, the uneven force of the support roller bearing of the rolling mill is revealed. The deformation and stress distribution of each column of FCRB are analyzed when the support roller is loaded, which provides a theoretical basis for the establishment of an FCRB temperature field model.
(2) Based on the rolling mechanics model, the temperature calculation model of FCRB is established, and the temperature field of FCRB is calculated using the finite element method.
(3) The overall temperature distribution of FCRB is analyzed, and the inhomogeneity of the FCRB temperature under uneven load conditions is revealed. The temperature change trend of FCRB under different load and speed conditions is further analyzed.
(4) Based on the rolling mill comprehensive fault simulation test bench, the temperature distribution test experiment was carried out to verify the accuracy of the temperature calculation model.

The remainder of this article is organized as follows. In Section 2, the mechanical modeling calculation and analysis of four-row roller bearings are carried out. In Section 3, we calculate and analyze the temperature field of the four-row roller bearing of the rolling mill. In Section 4 the comprehensive test simulation test bench of rolling mill is designed and built, and the FCRB temperature experiment is carried out. Conclusions are drawn in Section 5.

## 2. Mechanical Analysis Model of FCRB Based on Slicing Method

### 2.1. Mechanical Analysis of Bearing

In the mechanical analysis of bearings, the load contact problem of bearings is more complicated. In the rolling process, the rolling mill bearing bears a large load and the load distribution is uneven. In the rolling process, it is often affected by high load, high temperature, and uneven load, and the nonlinear model is difficult to establish. Based on the elastic contact theory, Harris [16] proposed a discrete bearing model processing method for bearing analysis called the “slice method”. Before using the slice method to analyze the stress state of bearings, the following assumptions must be made: (a) It is assumed that any contact between rollers and raceways can be divided into a certain number of “slices” in the parallel and radial planes. (b) It is assumed that due to the small contact deformation, the shear stress between the slices can be ignored, and only the contact deformation of the rolling element inside the bearing is considered.

**Figure 1:**
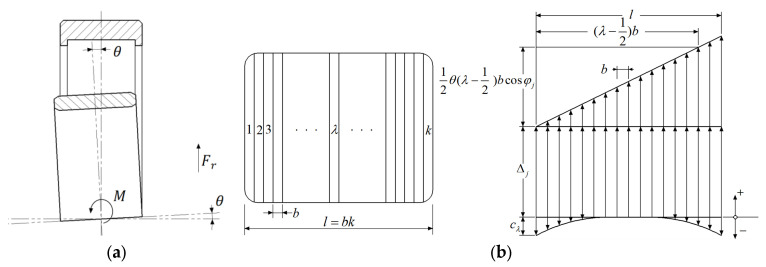
(**a**) Inclination of cylindrical roller bearing inner race. (**b**) Roller-raceway deformation caused by radial load, tilt, and crown.

As shown in Figure 1a, the inner ring of FCRB tilts relative to the outer ring. When the radial load acts on a single-row cylindrical roller bearing, the deformation of each slice of the roller with convexity can be divided into three components: (a) Deformation $\Delta_{j}$ caused by roller with radial load at azimuth $\varphi_{j}$. (b) Deformation $c_{\lambda }$ on the λ-th slice due to the convexity of the roller. (c) Bearing axis or roller tilt caused by the deformation of azimuth $\varphi_{j}$. A schematic of each component is shown in Figure 1b.

For the bearing inclination angle θ generated as shown in Figure 1a, at the roller with azimuth $\varphi_{j}$, the effective inclination angle is $\pm 2\theta cos\varphi_{j}$, where $0<\varphi_{j}<90{}^{\circ}$ is positive and $90{}^{\circ}<\varphi_{j}<180{}^{\circ}$ is negative. At this time, if the roller is divided into k slice and the width of each slice is *b*, the total deformation of the roller raceway at azimuth $\varphi_{j}$ and slice $k_{j}$ is

(1) $$\delta_{\lambda j}=\Delta_{j}\pm \frac{\theta }{2}\left(\lambda -\frac{1}{2}\right)b\cos\varphi_{j}-c_{\lambda }$$

**Figure 2:**
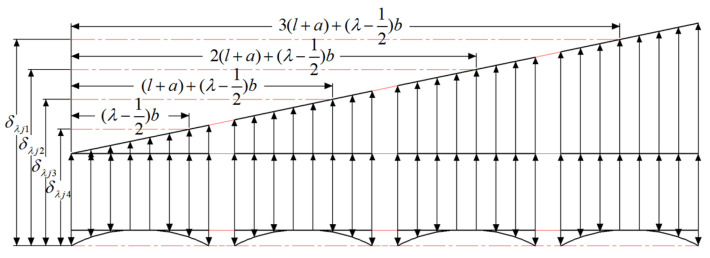
Deformation of rolling mill FCRB.

It is assumed that the deformation of FCRB is linearly distributed between rows [26]. The contact deformation between the roller and the raceway can be expressed as follows.

(2) $$\delta_{\lambda ji}=\Delta_{j}\pm \frac{\theta }{2}\left[\left(4-i\right)\left(l+a\right)+\left(\lambda -\frac{1}{2}\right)b\right]\cos\varphi_{j}-c_{\lambda }$$

where i represents the number of rows of FCRB and is an integer from 1 to 4. a represents the distance between the rolling elements of a FCRB, and l represents the length of the bearing roller. The contact deformation of the roller with the raceway is shown in Figure 2.

Based on the relationship between the load on the bearing roller and the radial displacement generated by the roller raceway, Palmgren [27] summarized the following contact deformation formula

(3) $$\delta =3.84\times 10^{-5}\frac{Q^{0.9}}{l^{0.8}}$$

where *Q* represents the normal load on the bearing roller and raceway. Since the roller is divided into *k* slices, since the width of each slice is *b*, the contact length is l. Let l=bk, q=Q/l, then Equation (3) becomes

(4) $$\delta =3.84\times 10^{-5}q^{0.9}{\left(bk\right)}^{0.1}$$

where q is the uniform load generated by the roller on the contact length. Equation (4) can be changed into the following equation.

(5) $$q=\frac{\delta^{1.11}}{1.24\times 10^{-5}{\left(bk\right)}^{0.11}}$$

Take Equation (2) into Equation (5) to obtain

(6) $$q_{\lambda ji}=\frac{{\left\{\Delta_{j}\pm \frac{\theta }{2}\left[\left(4-i\right)\left(l+a\right)+\left(\lambda -\frac{1}{2}\right)b\right]\cos\varphi_{j}-c_{\lambda }\right\}}^{1.11}}{1.24\times 10^{-5}{\left(bk\right)}^{0.11}}$$

where $q_{\lambda ji}$ is the radial load borne by the λ-th slice element of the roller at azimuth $\varphi_{j}$. Whether all rollers in the contact area and the slices of the raceway are subjected to the load depends on the bearing load and the inclination of the inner race. The total load on the roller at azimuth $\varphi_{j}$ is

(7) $$Q_{ji}=\frac{b^{0.89}}{1.24\times 10^{-5}k^{0.11}}\overset{i=4}{\underset{i=1}{\sum }}\overset{\lambda =k}{\underset{\lambda =1}{\sum }}{\left\{\Delta_{j}\pm \frac{\theta }{2}\left[\left(4-i\right)\left(l+a\right)+\left(\lambda -\frac{1}{2}\right)b\right]\cos\varphi_{j}-c_{\lambda }\right\}}^{1.11}$$

To determine the load of each roller, the static balance equation must be satisfied. The load of the bearing shall satisfy the following equation

(8) $$\frac{F_{r}}{2}-\overset{i=4}{\underset{i=1}{\sum }}\overset{j=\frac{Z}{2}+1}{\underset{j=1}{\sum }}\tau_{j}Q_{ji}\cos\varphi_{j}=0\{\begin{array}{c} \tau_{j}=0.5;\varphi_{j}=0,\pi \\ \tau_{j}=1.0;\varphi_{j}\neq 0,\pi \end{array}$$

Take Equation (7) into Equation (8) to get

(9) $$\frac{0.62\times 10^{-5}F_{r}}{b^{0.89}}-\overset{i=4}{\underset{i=1}{\sum }}\overset{j=\frac{Z}{2}+1}{\underset{j=1}{\sum }}\frac{\tau_{j}\cos\varphi_{j}}{k^{0.11}}\overset{\lambda =k}{\underset{\lambda =1}{\sum }}{\left\{\Delta_{j}\pm \frac{\theta }{2}\left[\left(4-i\right)\left(l+a\right)+\left(\lambda -\frac{1}{2}\right)b\right]\cos\varphi_{j}-c_{\lambda }\right\}}^{1.11}=0$$

For the overturning moment acting on the same plane inside the bearing, the following equilibrium conditions shall be satisfied:

(10) $$\frac{M}{2}-\overset{i=4}{\underset{i=1}{\sum }}\overset{j=\frac{Z}{2}+1}{\underset{j=1}{\sum }}\tau_{j}Q_{ji}e_{ji}\cos\varphi_{j}=0\{\begin{array}{c} \tau_{j}=0.5;\varphi_{j}=0,\pi \\ \tau_{j}=1.0;\varphi_{j}\neq 0,\pi \end{array}$$

where $e_{ji}$ denotes the eccentricity of the load at each position of the roller, derived from the following formula:

(11) $$e_{ji}=\frac{\sum_{\lambda =1}^{\lambda =k}q_{\lambda ji}\left(\lambda -\frac{1}{2}\right)b}{\sum_{\lambda =1}^{\lambda =k}q_{\lambda ji}}-\frac{l}{2}$$

Take Equations (7) and (11) into Equation (10) to obtain

(12) $$\frac{0.62\times 10^{-5}M}{b^{0.89}}-\overset{i=4}{\underset{i=1}{\sum }}\overset{j=\frac{Z}{2}+1}{\underset{j=1}{\sum }}\frac{\tau_{j}\cos\varphi_{j}}{k^{0.11}}\overset{\lambda =k}{\underset{\lambda =1}{\sum }}{\left\{\Delta_{j}\pm \frac{\theta }{2}\left[\left(4-i\right)\left(l+a\right)+\left(\lambda -\frac{1}{2}\right)b\right]\cos\varphi_{j}-c_{\lambda }\right\}}^{1.11}\left[\left(\lambda -\frac{1}{2}\right)b-\frac{l}{2}\right]=0$$

**Figure 3:**
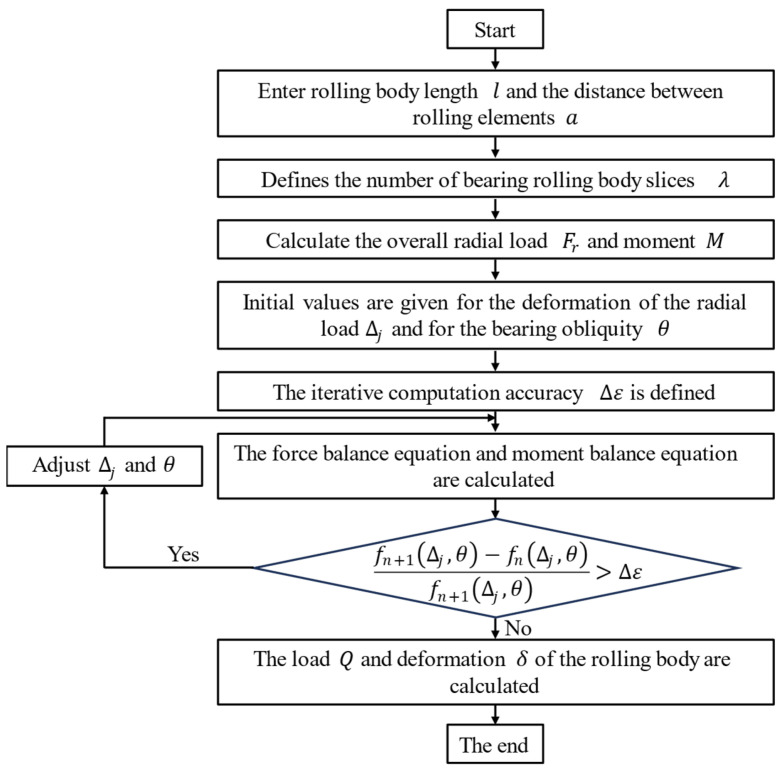
The flow chart for calculating the load distribution of the bearing.

Figure 3 shows the flow chart for calculating the load distribution of the bearing. The Newton–Raphson method (NRM) is a common method of calculating a system of nonlinear equations. In the figure, the Newton iterative method is used to calculate the system of linear equations consisting of Equations (9) and (12), the rolling body load, contact deformation, and overall load distribution of rolling mill FCRB. The calculation process of this method is as follows: first, the geometric parameters of the FCRB, such as roller length l and the distance between rolling elements a, are entered, and the number of slices of the roller λ is defined. The overall radial load $F_{r}$ and moment M of the FCRB of a single rolling mill are calculated by the rolling pressure of the rolling mill. The deformation ${∆}_{j}$ and bearing inclination θ caused by the radial load are given initial values. At the same time, the calculation precision of iteration is defined as ∆ε. Define the system of linear equations that consists of Equations (9) and (12), and bring in ${∆}_{j}$ and θ to enter the loop. By controlling the error accuracy value ∆ε, when the result does not converge, ${∆}_{j}$ and θ are adjusted. The iteration ends when the convergence is stable. Finally, the contact deformation δ of the rolling element can be obtained by introducing ${∆}_{j}$ and θ into Equation (2), and the load *Q* of the single rolling element of the bearing can be obtained by introducing Equation (7).

### 2.2. Rolling Mill Bearing Load Distribution Calculation

In this paper, the 1140 mm six-high cold strip mill is used as the calculation object of bearing load distribution. The mill can roll steel with a maximum width of 1140 mm, and the FCRB used in the backup roll plays a major supporting role in rolling.

#### 2.2.1. Finite Element Modeling and Meshing

This paper uses SolidWorks 2022 to model the experimental rolling mill, which consists of two bearing housings, two sets of multi-row roller bearings, and rolls. In addition to the assumptions of Hertz elastic contact theory, there are also the following assumptions: (a) Each component of the bearing is an isotropic material, and the influence of carburizing and heat treatment processes on its elastic modulus and Poisson’s ratio is not considered. (b) The cage does not influence the radial load on the bearing, so the cage is omitted, and the rolling components and the rings are relatively static. (c) To simplify the calculation, redundant chamfers, fillets, threaded holes, and other structures irrelevant to the analysis are removed in the simulation process.

**Figure 4:**
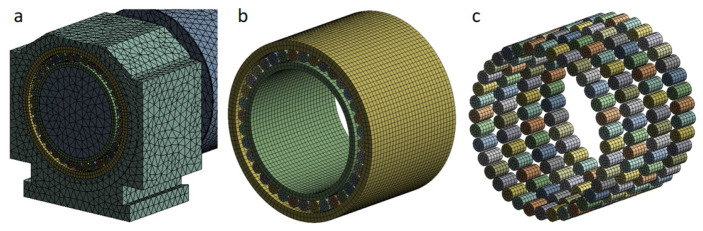
Rolling mill-bearing grid division model: (**a**) The overall grid of the model. (**b**) Bearing grid. (**c**) Roller body grid.

The 8-node tetrahedron element and the 8-node hexahedron element are used to divide the mesh as a whole. The mesh has a subdivision of the contact surface between the roller and the ring, the bearing seat and the surface of the outer ring, and the rotating shaft and the surface of the inner ring. The number of the mesh is 197,840, and the number of nodes is 524,853. The rolling mill-bearing grid division model is shown in Figure 4.

#### 2.2.2. Boundary and Load Settings

Add constraints: According to the operational characteristics of rolling mill bearings, the bearing seat of this equipment is fixed by the frame. In the real operational process, the rings of the FCRB of the rolling mill adopt an interference fit with the roll and the bearing seat. Therefore, in the FEM, the surfaces of rings of FCRB are set in bonded contact with the rollers and the chock. At the same time, the contact between the rings and the rollers is set as friction. The radial force on the cylindrical surface is set on the surface of the backup roll in the axial direction to simulate the inter-roll pressure on the backup roll during the rolling process.

**Figure 5:**
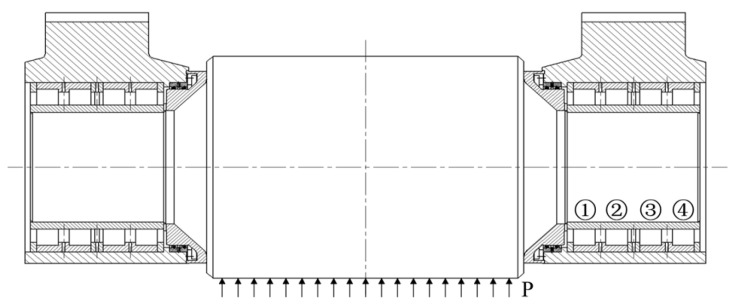
Support roller force diagram.

The force state is given away in Figure 5. To smooth the progress of the analysis, a row of bearings close to the support roller is selected as the first row, a row of bearings near the transmission side is selected as the fourth row, and the two rows of bearings in the middle are the second row and third row. The specific structural and material parameters of the used bearings are shown in Table 1.

#### 2.2.3. Results and Analysis

**Figure 6:**
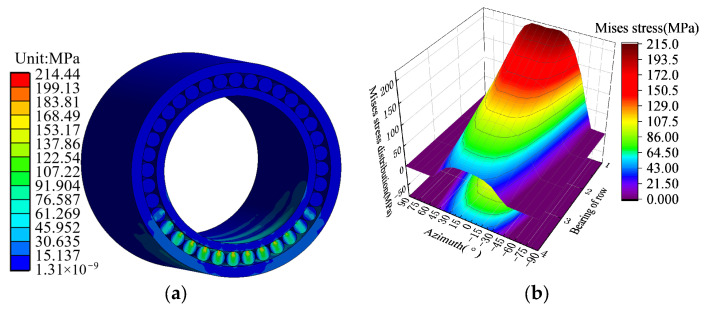
(**a**) Mises stress distribution of bearing. (**b**) The maximum stress distribution of rollers.

When the roll is subjected to 1000 t inter-roller pressure, FCRB produces corresponding stress and deformation. Through the mechanical simulation of the FCRB and roller system, the Mises stress distribution of the FCRB as shown in Figure 6a is obtained. Figure 6b shows the maximum stress distribution of the FCRB roller, and the stress distribution trend is consistent with the stress distribution trend shown in Figure 6a. At the same time, the deformation of the FCRB is linearly distributed between the rows.

**Figure 7:**
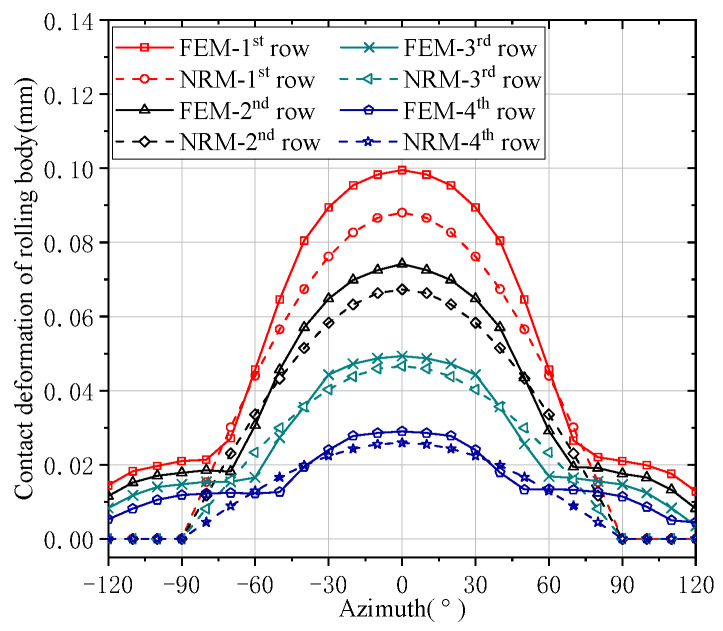
Contact deformation of the rolling body.

**Figure 8:**
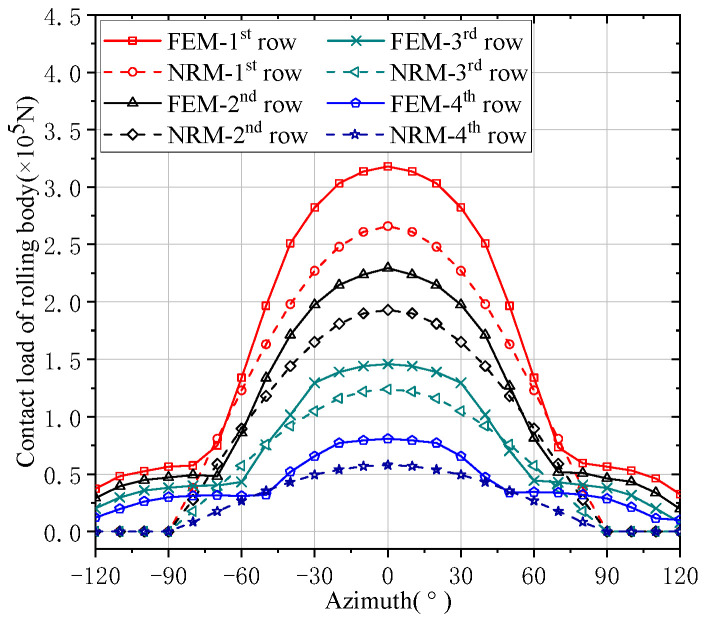
Contact load distribution of the rolling body.

The contact deformations and load distributions of FCRB rollers are shown in Figure 7 and Figure 8. Although the results of FEM and NRM are different, the difference between them is not significant. It can be found from Figure 7 and Figure 8 that each row of FCRB generally conforms to the distribution law of the bearing load in the Harris model, that is, the load on the rolling elements of the bearing with different azimuth angles is related to its azimuth angle [24]. The first row of numerical comparisons in which the contact deformation of the bearing roller differs most from the load is shown in Table 2. The result error of contact deformation and contact load is 11.42% and 16.35%, respectively. As can be seen from the figure, the trend of the results calculated by NRM is similar to that calculated by FEM. The contact deformation and load of the rolling body decrease in turn. The contact load on the first row is the largest and the contact deformation is the largest; the contact load on the fourth row is the smallest and the contact deformation is the smallest. As shown in Figure 8, the load on the first row of the roller is the largest, reaching 35% of the overall bearing load, compared with the other rows of the roller. The load on the other rows of the bearing is less. Since the working life of the entire bearing should be considered in terms of the row with the largest load, non-uniform load will result in a significant reduction in the life of a FCRB. 

## 3. Temperature Field Calculation and Analysis of FCRB

### 3.1. Modeling of Heat Generation in FCRB

In addition to using mathematical methods to calculate and analyze the friction torque of rolling bearings, Palmgren [27] obtained an experiential equation for calculating the friction torque of bearings through tests on numerous types and sizes of bearings. Since the radial force is much more prominent in the FCRB of the rolling mill, the following mainly analyzes the friction torque produced by the radial force of the bearing. In the Palmgren calorific value calculation model, the total friction torque is

(13) $$M=M_{l}+M_{v}$$

In the formula, $M_{l}$ is the friction torque caused by the external load. $M_{v}$ is the friction torque generated when the rolling element passes through the viscous lubricant in the bearing cavity. M is the friction torque by the load and the viscous lubricant. For the $M_{l}$, the following formula can be used for calculation [16,27].

(14) $$M_{l}=f_{1}F_{\beta }d_{m}$$

In the formula, $f_{1}$ is the bearing load friction coefficient. For a radial roller bearing with a cage, $f_{1}$= 0.0002–0.0004. $F_{\beta }$ is radial load. $d_{m}$ is the pitch diameter of the bearing.

The fluid viscous friction torque of the lubricant is:

(15) $$M_{v}=\{\begin{array}{c} 10^{-7}f_{0}{\left(vn\right)}^{2/3}d_{m}^{3},vn\geq 2000\\ 160\times 10^{-7}f_{0}d_{m}^{3},vn<2000 \end{array}$$

In the formula, n is the bearing speed. $f_{0}$ is the coefficient of viscous friction. v is the kinematic gooeyness of the lubricant at the operational temperature.

Energy is equivalent to torque times speed or torque times rotational speed. Therefore, the energy loss caused by bearing friction can be expressed as:

(16) $$H=H_{l}+H_{v}=0.001M\cdot \omega$$

In the formula, $H_{l}$ and $H_{v}$ are the friction losses caused by the applied load and viscous friction, respectively. ω is the bearing angular velocity, the unit is rad/s. If the velocity is in r/min, Equation (16) can be changed to

(17) $$H=H_{l}+H_{v}=1.047\times 10^{-4}M\cdot n$$

Kannal and Barber [28] assume that the contact heat transfer between the rolling body and the rings is the friction heat generation of the moving heat source on the semi-infinite body; then, the heat flux on the contact face is

(18) $$q=\{\begin{array}{c} H_{c}/A_{c},t_{i}<t<t_{i}+\Delta t\\ 0,t_{i}+\Delta t<t<t_{i+1} \end{array}$$

where $H_{c}$ is energy loss. $A_{c}$ is the contact face. $t_{i}$ and $t_{i+1}$ are the time before and after the contact face. ∆t is the time of contact. The heat flux in the contact area can be expressed as heat per unit area [29]. Thus, Equation (18) can be transformed into

(19) q=H/S

where H is the friction loss generated in the movement. S is the whole contact zone of the movement.

Because the physical properties inside the bearing are the same or similar, assuming that the friction heat is conveyed on the contact surface in a ratio of 1:1 [30,31], the heat of each part of the bearing can be described as follows.

(20) $$H_{in}=H_{out}=\frac{1}{4}H$$

(21) $$H_{roller}=\frac{1}{2}H$$

where $H_{in}$, $H_{out}$ and $H_{roller}$ are the heat produced by the internal and external contact surface and rolling elements due to energy loss, respectively. H is the total heat caused by bearing friction.

As the rolling mill FCRB runs, the internal ring and the rollers rotate. It can be considered that heat is evenly distributed on the contact face of the inner ring and the roller. Therefore, the heat flux of the internal ring contact surface and the roller surface can be expressed as

(22) $$q_{in}=H_{in}/S_{in}$$

(23) $$q_{roller}=H_{roller}/S_{roller}$$

Among these, $q_{in}$ and $q_{roller}$ are the friction loss of the inner raceway and rolling element, respectively. $S_{in}$ and $S_{roller}$ are the heating area of inner raceway and rolling element, respectively.

The heat flow of the outer ring can be divided into heat flow $q_{outl}$ caused by load friction and heat flow $q_{outv}$ caused by viscous friction.

(24) $$q_{out}=q_{outl}+q_{outv}$$

Among them, the heat flux generated by the viscous friction of the external ring is

(25) $$q_{outv}=\frac{1}{4}H_{v}/S_{outv}$$

**Figure 9:**
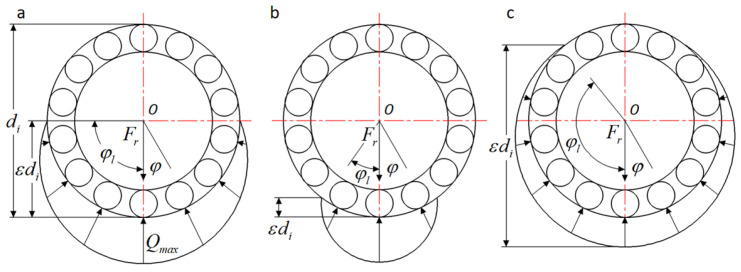
Load distribution of rolling elements at different clearances: (**a**) ε= 0.5, $\varphi_{l}=\pm 90{}^{\circ}$, zero clearance, (**b**) 0<ε<0.5, $0<\varphi_{l}<90^{{}^{\circ}}$, positive clearance, (**c**) 0.5<ε<1,$90^{{}^{\circ}}<\varphi_{l}<180^{{}^{\circ}}$, negative clearance and interference fit.

$S_{outv}$ is the heating area where viscous friction occurs on the outer ring. The load distributions of roller bearings [16] with different clearances are shown in Figure 9. The contact load $F_{\varphi }$ at azimuth φ can be expressed as

(26) $$F_{\varphi }=F_{max}{\left[1-\frac{1}{2\varepsilon }\left(1-cos\varphi \right)\right]}^{\frac{10}{9}}$$

where $F_{max}$ is the maximum contact load between the rollers and the outer ring face. ε is the diameter distribution factor to load. From these, ε = 0.5 for zero clearance; 0<ε<0.5 for positive clearance; 0.5<ε<1 for negative clearance or interference fit. Therefore, ε can be considered as the ratio of the projection of the load area on the bearing diameter to the diameter.

Since the heat flux generated by the load is related to load, Equation (26) can be expressed as Equation (27).

(27) $$q_{outl}=\{\begin{array}{c} q_{max}{\left[1-\frac{1}{2\varepsilon }\left(1-cos\varphi \right)\right]}^{\frac{10}{9}},\left|\varphi \right|\leq \varphi_{l}\\ 0,\left|\varphi \right|>\varphi_{l} \end{array}$$

where $q_{max}$ is the heat flow generated by the maximum load. From the geometric relationship, Equation (27) can also be transformed into

(28) $$q_{outl}=\{\begin{array}{c} q_{max}{\left[\frac{cos\varphi_{l}\;\pm \;cos\varphi }{1\;\pm \;cos\varphi_{l}}\right]}^{\frac{10}{9}},\left|\varphi \right|\leq \varphi \\ 0,\left|\varphi \right|>\varphi_{l} \end{array}$$

Among these, when the bearing has a positive clearance, the denominator of the equation is a negative sign; when the bearing has a negative clearance and an interference fit, the denominator of the equation is a positive sign. Due to the conservation of energy, the heat flux generated by the load on the outer ring must satisfy the following conditions:

(29) $$\int_{-\varphi_{l}}^{\varphi_{l}}q_{outl}\cdot l\cdot r\varphi d\varphi =\frac{1}{4}H_{l}$$

**Figure 10:**
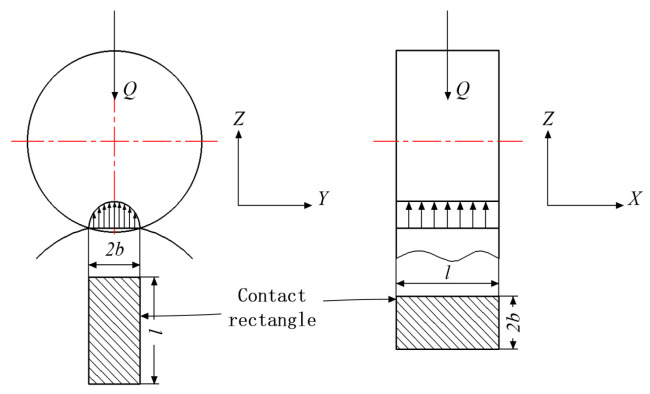
Schematic diagram of roller bearing contact.

Among these, as shown in Figure 10, *l* is the effective length of the contact between the roller and the ring. *r* is the radius of the outer ring contact face.

### 3.2. Internal Energy Transfer Model of Bearing

There are three main basic heat transfer models inside a bearing: heat conduction within a solid, heat convection between a solid and a surface liquid, and heat radiation between two substances separated from each other by space. Among these, the thermal radiation is slight, so it is ignored in the calculation.

#### 3.2.1. Heat Conduction Calculation

Heat conduction can be articulated as a linear function of the temperature difference in a solid, namely

(30) $$H_{c}=\frac{kS}{L}\left(T_{1}-T_{2}\right)$$

In the formula, S is used to calculate the area of heat conduction between two points. L is the heat transfer distance between two points. $T_{1}$ and $T_{2}$ are the real-time temperature at two points, *k* is the thermal conductivity inside the model.

#### 3.2.2. Heat Convection Calculation

Harris gave a formula for calculating the heat convection between the bearing and the oil:

(31) $$h_{v}=0.332kP_{r}^{\frac{1}{3}}{\left(\frac{u}{vx}\right)}^{\frac{1}{2}}$$

where the heat convection between the bearing and the oil is calculated; u is the surface velocity of the cage. For the heat convection between the inner face of the bearing seat and the oil, u is 1/3 of the cage, and x is the inner diameter of the housing. v is for kinematic viscosity. $P_{r}$ is the Plante number of the oil.

The heat convection between the outer surface of the housing and the air can be approximated by Equation (32):

(32) $$h_{a1}=\{\begin{array}{c} 0.3{\left(T-T_{a}\right)}^{\frac{1}{4}}\\ 0.3\frac{k_{a}}{D_{h}}R_{e}^{0.57} \end{array}$$

In the formula, $T_{a}$ is the ambient temperature of the surface of the bearing housing. $k_{a}$ is the thermal conductivity of the air. $D_{h}$ takes the diameter of the housing. $R_{e}$ is the Reynolds number of the bearing housing, expressed by the formula

(33) $$R_{e}=\frac{u_{a}D_{h}}{v_{a}}$$

where $u_{a}$ is the air speed and $v_{a}$ is the air kinematic viscosity.

The formula for the heat convection between the outer surface of the rotating shaft and the air is [32]

(34) $$h_{a2}=0.119\frac{k_{a}}{D_{h}}R_{e}^{\frac{2}{3}}$$

### 3.3. Simulation of FCRB Temperature

Based on the mechanical model of FCRB, the temperature field simulation analysis is carried out. The geometric parameters and working conditions of the bearing are input into the calculation model of heat production and heat transfer of FCRB constructed by MATLAB 2018a software, and the calculation results are further applied to each surface of the bearing to simulate it.

#### 3.3.1. Finite Element Boundary Condition Setting

Since the heat of the bearing system is generated by the friction between the rollers inside the bearing and the rings, the heat flux density is applied to the raceways of the rings and the contact surfaces of the rollers. The parameters of the lubricating oil used in the bearing are shown in Table 3.

#### 3.3.2. FCRB Temperature Distribution

**Figure 11:**
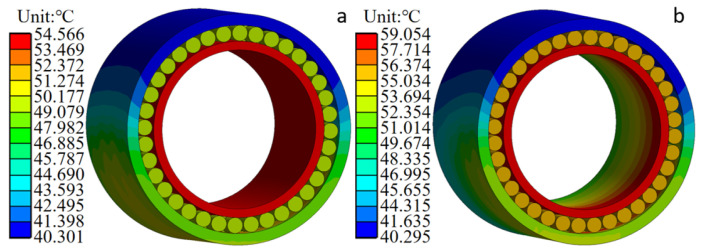
Cloud diagram of bearing temperature distribution: (**a**) The load is uniformly distributed; (**b**) the load is not uniformly distributed.

According to the working characteristics of the six-high cold rolling mill, the temperature of the bearing model is calculated under the uniform load and non-uniform load under the condition of rolling force 10,000 KN and rotation speed 100 rpm/min. The temperature distribution of FCRB as shown in Figure 11 is obtained by setting the ambient temperature to 30 °C and the internal oil temperature to 40 °C. In each row of FCRB, the average temperature of the internal raceway is higher than the rollers and the external raceway. This is because the external raceway has a larger heat dissipation area. At the same time, the average temperature of the internal raceway is upper because the centrifugal force causes the inside raceway lubricant to splash on the external raceway. Rolling mill bearings have more rollers, so the rollers have more contact area with the oil, resulting in greater heat dissipation area and lower temperature than normal bearings. In addition, a significant temperature change can be seen on the bearing outer ring surface. This is because of the force acting on the outer face in relation to the azimuth. The lesser the azimuth, the greater the contact force between the roller and the rings. The non-uniformity of the bearing outer ring temperature is caused by the non-uniformity of the bearing outer ring force.

#### 3.3.3. Temperature Analysis of Inner Ring of FCRB

**Figure 12:**
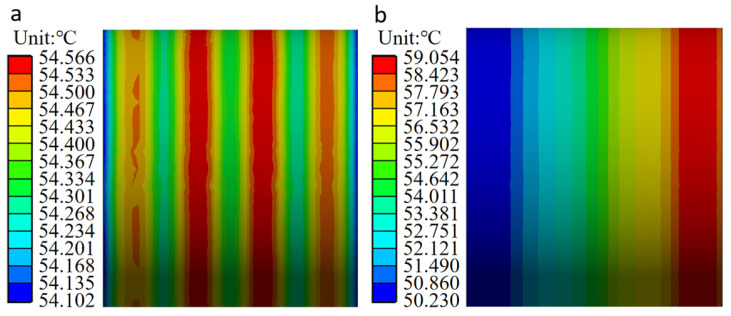
Axial temperature distribution cloud diagram of inner ring of FCRB (10,000 KN, 100 rpm): (**a**) The load is uniformly distributed; (**b**) the load is not uniformly distributed.

According to the temperature distribution in Figure 12, the temperature distribution of FCRB with uniform load distribution is uniform, the greatest temperature is mostly concentrated in the inner ring, and the highest temperature of the bearing is stable at 54.566 °C. The temperature distribution of FCRB under a non-uniform load is non-uniform, and the inner ring temperature at the position of the largest load is the highest, reaching 59.054 °C. The maximum temperature difference of the bearing is 4.488 °C under the condition of the uniform load. The maximum temperature difference of the bearing is 8.824 °C under the condition of a non-uniform load.

**Figure 13:**
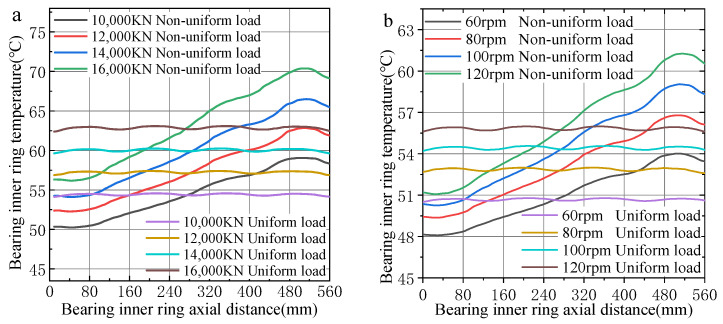
Axial temperature distribution of inner ring of FCRB: (**a**) Different loads; (**b**) different speeds.

Figure 13 shows the consequence of force and rotational rate on the temperature of the internal ring under two load conditions: uniform and non-uniform. Under the condition of uniform load, the temperature of the inner ring in the axial direction is relatively stable, and the temperature of the contact part between the inner ring raceway and the rolling element is higher than that of the non-contact part. The primary reason for this occurrence is that the contact part is the primary heat source. Under the same heat dissipation conditions, the temperature of the contact part is higher than the temperature of the non-contact part. Under the condition of non-uniform load, the temperature of the bearing inner ring along the axial direction generally shows a gradually increasing trend. The temperature trend is consistent with the bearing load trend. The temperature of the inner ring reaches the maximum value of the inner ring temperature in the row with the largest load. This also shows that the temperature distribution is related to the load distribution of the bearing.

**Figure 14:**
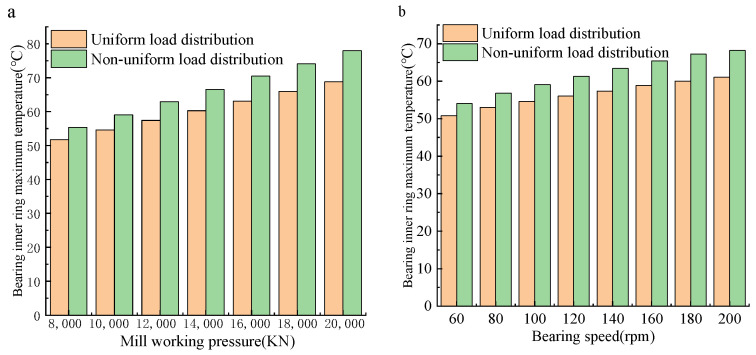
Maximum temperature change of bearing inner ring: (**a**) Different loads; (**b**) different speeds.

Figure 14 shows the result of load and speed on the maximum temperature of the inner ring of FCRB under two different bearing load distribution conditions. The temperature of the inner ring of the FCRB increases with the improvement of the force of the support roller, and the temperature growth rate of the FCRB with uneven force is significantly higher than that when the bearing is uniformly stressed. Therefore, under the same heat generation conditions, the FCRB subjected to a non-uniform load state will generate a larger local temperature.

#### 3.3.4. Temperature Analysis of Outer Ring of FCRB

**Figure 15:**
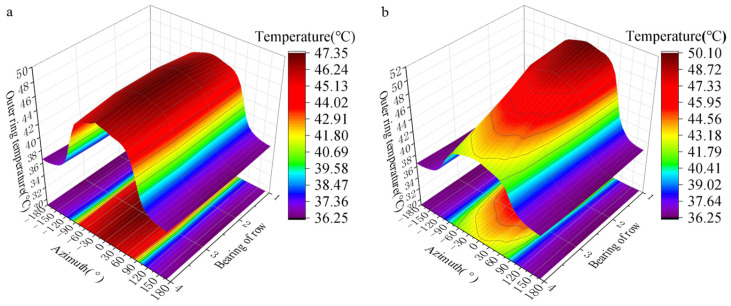
Bearing outer ring temperature distribution: (**a**) The load is uniformly distributed; (**b**) the load is not uniformly distributed.

The temperature distribution on the outer ring face under two different loading conditions is shown in Figure 15. Temperature of the outer ring is related to the azimuth angle. There is a negative correlation between the azimuth angle and temperature. The temperature reaches its maximum when the azimuth angle is 0°. The outer ring is uniformly distributed along the axial direction under an even load. At the same time, under the condition of non-uniform load, the temperature of the outer ring of FCRB increases along the axial route, which is caused by the increase in the load-bearing in the axial route.

**Figure 16:**
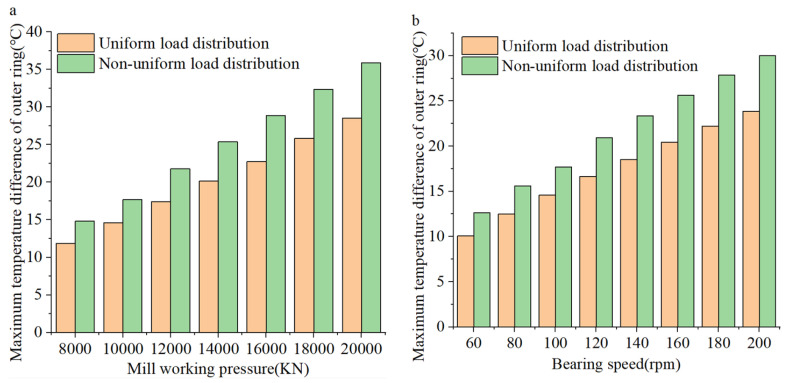
Maximum temperature difference of bearing outer ring: (**a**) Different loads; (**b**) different speeds.

Figure 16 shows the utmost temperature discrepancy of the outer ring under different load and speed conditions. With the rise in load and speed, the disparity between the maximum temperature and the minimum temperature of the outer ring of the FCRB keeps increasing. At the same time, the growth rate of the external ring temperature difference is higher when the FCRB load is unevenly distributed compared to when the load is evenly distributed. That is to say, the temperature discrepancy of the outer ring caused by the uneven load will be more obvious.

## 4. Experimental Testing and Result Analysis

### 4.1. Experimental Design

**Figure 17:**
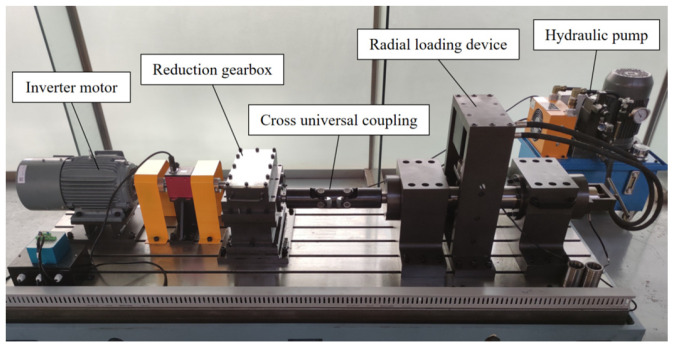
Rolling mill comprehensive test simulation test bench.

In order to study the temperature characteristics of the FCRB of the rolling mill and prove the precision of the temperature calculation model, the following comprehensive test simulation test benches for the rolling mill were designed and built to conduct the FCRB temperature experiment, as shown in Figure 17. The test bench consists of a frequency conversion motor, radial loading device, axial loading device, reduction gearbox system, roll bearing system, etc. The test bench can test physical attributes such as rotational speed, spindle torque, bearing vibration, shaft force, bearing temperature, etc. The experimental bench is powered by a frequency conversion motor and is connected to a parallel shaft gearbox through an elastic coupling for speed change. The radial loading device of the roll adopts a hydraulic pump to exert a radial force on the rotating shaft, and the hydraulic cylinder’s maximum pressure is 20 KN.

The temperature sensor used in the experiment is composed of a thermocouple test line and a corresponding temperature signal acquisition device. The thermocouple temperature measurement line adopts the T-type thermocouple. Although the T-type thermocouple has a small measuring range and can be tested at a maximum temperature of not more than 300 °C, it has high accuracy, high sensitivity, and low price. A 0.05 mm thin film temperature probe can be made from the thermocouple temperature measuring line, which can measure the temperature of the object surface in a small test space and can measure the relatively stable temperature signal. Therefore, the T-type thermocouple is selected as the temperature measurement element of the temperature test. At the same time, the multi-channel temperature signal acquisition device is used to collect the signals from the test line of the thermocouple. To ensure the accuracy of the experiment, the multi-channel temperature signal acquisition instrument is used to collect the temperature signals of many positions in a fixed working condition. The temperature signal collected by the signal acquisition device can reach 0.5 s and the temperature difference measured is within 0.2 °C; it can be connected to a computer through a serial port for real-time upload of the corresponding temperature data storage and analysis for the testers. Because of the advantages of the sensor in size and performance, the thermocouple sensor was selected as the temperature sensor of FCRB temperature test experiment.

**Figure 18:**
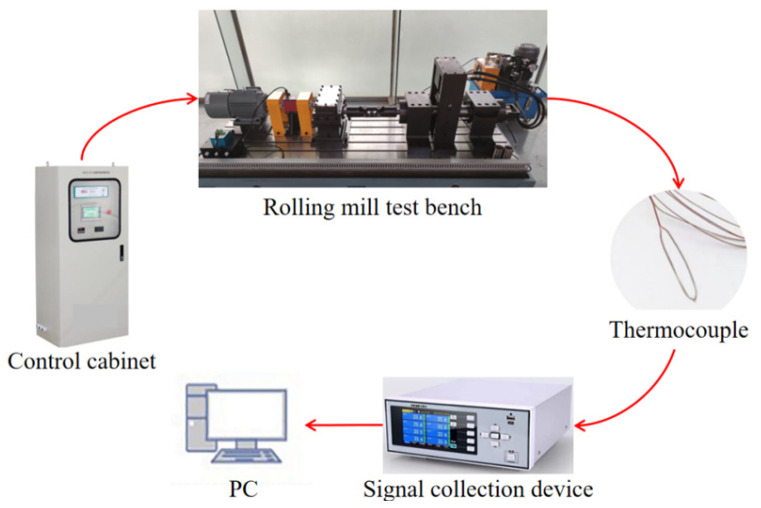
Schematic diagram of experimental test plan.

In this experiment, a FCRB with an inner diameter of 70 mm, an external diameter of 115 mm, and a width of 140 mm produced by Harbin Bearing Factory was used. As shown in Figure 18, the overall experimental scheme of the experiment is as follows: the control cabinet controls the rotating speed of the motor of the test worktable and the load applied by the hydraulic cylinder. The temperature-measuring finish of the thermocouple is attached to the surface of the outer ring to test the temperature of the outer ring of FCRB. The signal collection apparatus collects the temperature signal of the thermocouple test. The test signal is uploaded to the host computer for display and storage.

**Figure 19:**
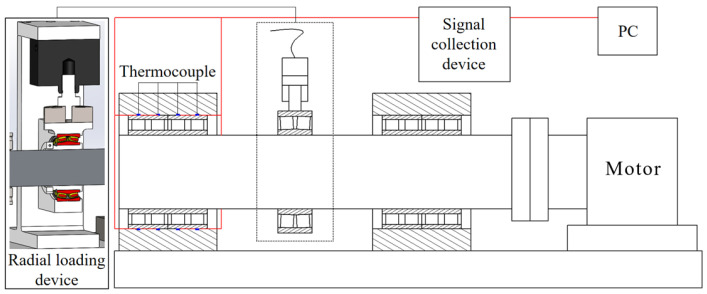
Schematic diagram of the experiment.

At the beginning of each test, the inverter motor operates at a fixed speed while the radial loading device continuously applies a fixed load to simulate the rolling process. The temperature signal collecting device is connected to the thermocouple temperature measuring line. The end of the thermocouple temperature measuring line is attached to the outer face of the outer ring for temperature measuring. In addition, the sign output end of the temperature sign acquisition device is connected to the upper computer, and the temperature sign is transmitted to the computer for display and storage in real time. Figure 19 shows the temperature test diagram for FCRB.

**Figure 20:**
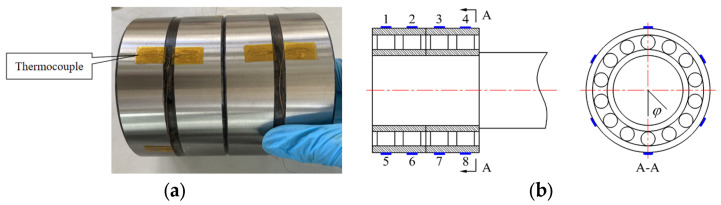
(**a**) FCRB surface thermocouple, (**b**) FCRB axial and circumferential sensor placement.

As exposed in Figure 20a, we attach the temperature-measuring end of the thermocouple to the outer ring, extend the thermocouple wire to the outside of the housing through the bearing groove, and connect the signal-collecting device at the end. The temperature-measuring end of the thermocouple is attached to each row of FCRB in turn along the axial direction. At the same time, four thermocouples are attached at the 180° symmetrical position. As shown in Figure 20b, eight thermocouples were placed in the axial direction of each row of the bearing to test the axial temperature distribution of each row of the bearing; six thermocouples were placed in the circumferential direction of the first row of the bearing (φ=0,60°,−60°,120°,−120°,180°, respectively) to test the circumferential temperature distribution.

### 4.2. Analysis of Experimental Results

**Figure 21:**
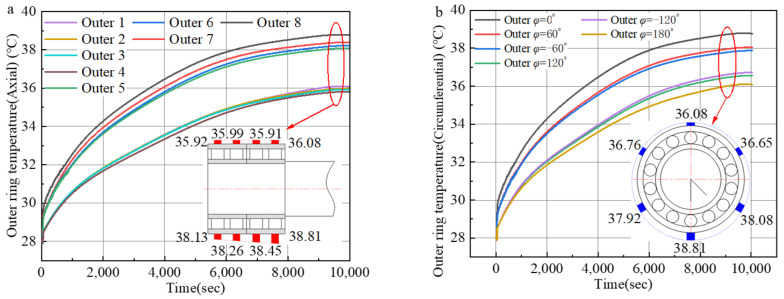
Temperature change of bearing outer ring (F = 10,000 N, *n* = 1500 rpm): (**a**) Axial temperature; (**b**) circumferential temperature.

As exposed in Figure 21, the temperature of the bearing outer race increases with time. In the early stage of operation, the temperature of the external ring of the FCRB is uniform, and the temperature difference at each test point is not obvious. Subsequently, the temperature of the external ring of the FCRB rose sharply, and the temperature difference at each point gradually increased. This is because, in the early stage of operation of the bearing, its calorific value is greater than the heat dissipation, causing its temperature to rise continuously. Eventually, the temperature difference at each point gradually stabilizes. At this time, the calorific value at each temperature measuring point is close to the heat dissipation, and the FCRB temperature is gradually maintained at a stable value. At this time, the bearing temperature reaches a steady state.

**Figure 22:**
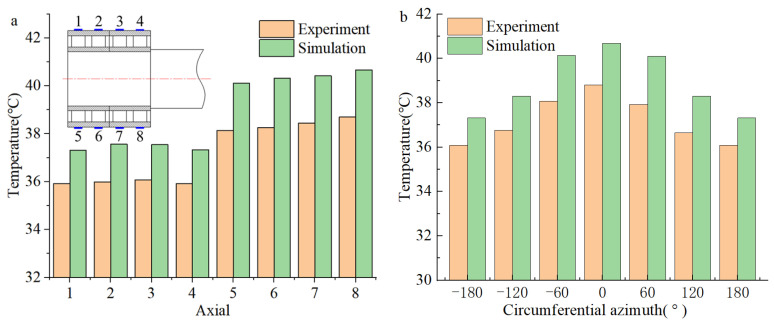
Bearing outer ring temperature distribution (S1: F = 10,000 N, *n* = 1500 rpm): (**a**) Axial temperature; (**b**) circumferential temperature.

**Figure 23:**
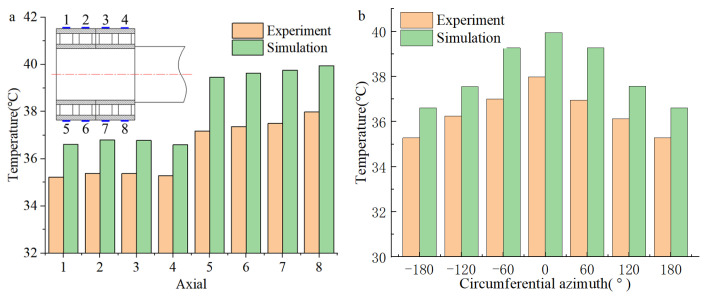
Bearing outer ring temperature distribution (S2: F = 20,000 N, *n* = 1000 rpm): (**a**) Axial temperature; (**b**) circumferential temperature.

As shown in Figure 22 and Figure 23, comparing the axial temperature of the outer bearing ring, the temperature in the load-bearing area is higher than that in the non-load-bearing area. At the same time, in the axial direction, the closer to the roll (the side where the force is applied), the higher the temperature of the load-bearing zone; the circumferential bearing temperature reaches its maximum value when the azimuth angle is zero. That is to say, in the axial direction, the temperature of the FCRB is positively correlated with the load, and the temperature reaches the maximum value in the row with the most considerable load. In the circumferential direction, the azimuth angle negatively correlates with temperature [24,33]. Due to the operational characteristics of the rolling mill, when the equipment is working, the force acts on the roll, resulting in uneven pressure on the four rows of roller bearings. This phenomenon leads to the inhomogeneity of the temperature. Therefore, higher temperatures are generated in the load-bearing zone of the row of bearings near the rolls. In addition, in the axial direction of the outer ring of FCRB, the average error of the experimental and simulated temperature results is 1.7 °C (4.3%). In the circumferential direction, the average error of the experiment and simulation is 2.2 °C (5.7%). Although there is a certain error, the error results are all within 10%, and the simulation results have a consistent trend with the experimental results.

## 5. Conclusions

In this paper, the temperature calculation model of FCRB is established by calculating and analyzing the mechanical model of FCRB. The temperature calculation model was verified by experiments. The conclusions are summarized as follows:

(1) The mechanical analysis of FCRB was carried out based on the slice method, and the mechanical model of FCRB was established by NRM and FEM. The mechanical analysis shows that the results of NRM and FEM are consistent in calculating the load distribution of FCRB. The calculation errors of contact deformation and contact load are 11.42% and 16.35%, respectively. It can be seen that the load on the first row of rollers is the largest, reaching 35% of the total load of the bearing.
(2) Based on the mechanical model of FCRB, the temperature calculation model of FCRB is established by the finite element method. The experiment verifies the accuracy of the simulation results, and the error between the experimental results and the simulation results is within 10%.
(3) Under non-uniform load conditions, the temperature of the FCRB outer ring is not only uneven in the circumferential process of the bearing, but also uneven in the axial process of the bearing. The uneven distribution of load is mainly the cause of uneven temperature. In the axial direction, the temperature of the FCRB is positively correlated with the load, and the temperature reaches the maximum in the row with the largest load. In the circumferential direction, the temperature is negatively correlated with the absolute value of the azimuth angle.
(4) Compared with the temperature distribution of FCRB under non-uniform load conditions, the temperature distribution of FCRB under uniform load conditions is more uniform. At the same time, with the increase of bearing load and rotational speed, the growth rate of ring temperature difference under uniform load is lower than that under non-uniform load. Therefore, in the case of uniform load distribution, the local temperature of the bearing can be avoided to a certain extent, which provides a reference for the design and application of the bearing.

## Figures and Tables

**Table 1:** Specific material parameters and structural parameters of FCRB.

Bearing Parameters	Value	Bearing Parameters	Value
Bearing inner diameter *d* (mm)	550	Young’s modulus (MPa)	2.19 × 10^5^
Bearing outer diameter *D* (mm)	800	Poisson’s ratio	0.3
Bearing diameter *d_m_* (mm)	665	Density (kg/m^3^)	7830
Bearing width *B* (mm)	85	Thermal conductivity (W/(m·°C))	50
Rolling body diameter *d_w_* (mm)	55	Specific heat capacity (J/(kg·°C))	460
Roller width *l* (mm)	85	-	-
Number of rolling elements in a single row *Z*	36	-	-

**Table 2:** Comparison between the results of FEM and NRM.

Parameter	FEM	NRM	Error
Deformation (mm)	0.09952	0.08815	11.42%
Contact load (N)	317,978	266,000	16.35%

**Table 3:** Bearing lubricating oil parameters.

Parameters	Unit	Value
Kinematic viscosity	mm^2^/s	2.19 × 10^5^
Density	kg/m^3^	0.3
Thermal conductivity	W/(m·°C)	7830
Specific heat capacity	J/(kg·°C)	50

## Data Availability

The experimental data can be obtained on request from ghs1993@163.com (H.G.). The experimental data is obtained through the National Engineering Research Center for Equipment and Technology of Cold Rolled Strip of Yanshan University. The experimental results are reproducible.
